# The Importance of Self-Selection and Childcare Leave Length for Child Penalty

**DOI:** 10.1007/s10680-024-09726-2

**Published:** 2025-01-15

**Authors:** Eva Österbacka, Tapio Räsänen

**Affiliations:** 1 Faculty of Social Sciences, Business and Economics and Law, Åbo Akademi University, Turku, Finland; 2 Present Address: Social Insurance Institution of Finland, Helsinki, Finland

**Keywords:** Child penalty, Earnings, Employment, Selection, Gender

## Abstract

Childbirth has consequences for mothers’ labour market outcomes which in turn has consequences for gender differences in pay. In the Finnish context, earnings-related parental leave can be extended with home care allowance which enables mothers to choose their childcare leave length with varying benefit levels. We empirically test the importance of choice of childcare leave length for the subsequent child penalty. We apply Finnish register data with information on the length of childcare leave at the individual and workplace levels. By using workplace comparisons, we can account for some of the endogeneity in choices of workplace, parity, and childcare leave length. By instrumenting the leave length with varying home care allowance levels, we categorise always-takers, never-takers, and compliers. We find that the child penalty is heterogeneous and highly related to the choice of leave length. Always-takers use longer leaves than the workplace average and are penalised, while never-takers use shorter leaves than the workplace average and experience only a mild child penalty. Compliers adjust their leave lengths to the allowance level and experience child penalties in between. These results support that self-selection of childcare leave length has implications for post-birth outcomes and in addition, that family policy can affect the child penalty and the gender gap in pay.

## Introduction

Gender gaps have slowly decreased over the last decades but are still persistent in economic life. The Nordic countries are considered comparatively gender equal. However, gender gaps in pay are still noticeable (Albrecht et al., 2015; Angelov et al., 2016; Bütikofer et al., 2018; OECD, 2018).

Gender differences in human capital have diminished over time, and differences in observable characteristics, such as education and other types of training, can explain only a small part of the gender gap in pay. Instead, a more decisive part can be explained by gender differences in rewards to characteristics, usually called the unexplained gender gap. Kleven et al. (2019b) argue that the importance of child-related inequalities for gender inequalities has increased.

Career breaks, reduced labour force participation, transfers to more family-friendly careers, reduced likelihood of promotion, or reduced hourly wage rates are all related to the labour market consequences of childbirth for women. These factors contribute to the gender gap caused by childbirth, also called the child penalty (Adda et al., 2017; Kleven et al., 2019b; Lucifora et al., 2021). Mothers’ choices may contribute to these changes or employers may discriminate mothers.

All in all, childbirth has consequences for mothers’ labour market outcomes. Previous population-level estimates of child penalties provide information on the average effects of labour market outcomes after childbirth. They generally miss heterogeneous effects and finer mechanisms, which cannot be observed from the population average.

In this paper, we empirically test the importance of childcare leave length for the subsequent child penalty. Previous research shows that extensions to short or moderate parental leaves are beneficial for mothers and children, while the research on extensions to long leaves is inconclusive (see e.g. Canaan et al., 2022). Our contribution to the previous child penalty literature is to emphasise the childcare leave length and, in particular, the significance of self-selection in a context where the leave length can be up to 3 years per child. Some mothers are more family-oriented, while others are more work-oriented which influence their labour market outcomes post-birth. Family-oriented are more likely to take care of their children for longer periods than work-oriented mothers. These choices have consequences for their earnings.

Empirically, the causal effects of variations in childcare leave lengths are difficult to estimate since choices are related to individual preferences, which cause endogeneity issues. In addition, some of the choices are influenced by workplace norms. By introducing workplace comparisons in our empirical contribution, we can control for some of the endogeneity. Furthermore, we apply an instrumental variable technique to clarify the effects of individual choices and varying replacement levels for different lengths of childcare leaves.

Our data set is extensive. Previous research has used quite narrow data sets when studying the workplace context. For instance, Wilner (2016) uses data from the private sector, while Lucifora et al. (2021) use data from one firm. We use representative Finnish register data covering both the private and public sectors. Our sample gives birth to their first child in 2002–2006. They are followed 5 years prior to and 10 years after their first childbirth. In addition to information on individual childcare leave length, we can extract information from their peers and calculate workplace averages. In the Finnish context, mothers can extend their earnings-related childcare leave by a flat-rate home care allowance. However, some municipalities offer an addition to the flat-rate home care allowance. These variations in allowances influence the childcare leave lengths. By utilising municipal variations as an instrument, we are able to empirically estimate the impact of childcare leave length on the child penalty.

Our results show that longer childcare leaves than the workplace average level increase the child penalty. Similarly, shorter than average childcare leaves reduce the child penalty. By using exogenous variation in the childcare leave length, we find support for a self-selection effect on different childcare leave lengths which has implications for post-birth labour market outcomes. Moreover, we provide evidence that family policy affects the child penalty and the gender pay gap.

## Related Literature and Institutional Context

### Background

Gendered childcare obligations may cause self-selection into different careers, both pre- and post-birth, which has consequences for women’s earnings. Uncertainty related to women’s childcare leaves contributes to employers’ deviating treatment of women and men. Human capital may also depreciate during childcare leaves, which in addition contributes to the career development. Below, we summarise how childcare leaves are related to self-selection, asymmetric information, discrimination, and human capital accumulation, and their labour market consequences for employed women.

Theoretically, total household income increases if family members specialise in different tasks (Becker, 1981, 1985). Complete gender specialisation pre-birth is not that widespread in contemporary societies. But, post-birth specialisation contributes to gender segregation and wage differentials through reduced work hours or transfer to more family-friendly careers (Adda et al., 2017; Kleven et al., 2019b). Consequently, mothers spend more time in effort-intensive childcare, while fathers spend more time in paid work. If mothers endogenously sort into workplaces more compatible with family life and with lower earnings, this (self-selection) sorting mechanism will contribute to the child penalty (Hotz et al., 2018).

Women make different choices though, and Hakim (2000), Hakim (2006) draw on preference theory, in explaining the heterogeneous choices women make. Work-centred women are committed to work, while home-centred are committed to their families. The largest group of women, adaptive women, prefer both a family and a career. Family policies can facilitate home-centred women’s choices, while work-centred women are not affected. The adaptive group of women, on the other hand, react, and adapt, to family policies. Kangas and Rostgaard (2007) test the theory empirically and conclude that family policies are important for childcare leave lengths and labour market attachment among mothers. Kleven et al. (2024) argue, on the other hand, that family policies do not contribute to reductions in the gender pay gap.

The gendered labour market contributes to the gender gap in pay (Adsera & Querin, 2023; Bertrand, 2020; Blau & Kahn, 2017; Sin et al., 2022). Women with more children tend to work in less-prestigious workplaces (Cools et al., 2017). Wilner (2016) argues, on the contrary, that the gender gap does not seem to stem from workplace selection effects, at least not among mothers. However, childless women and future mothers have similar careers until children are born, but mothers are less likely to be promoted than childless women in the same workplace (Lucifora et al., 2021). The public sector is generally regarded as more family friendly than the private sector. The earnings penalty seems to be lower in the public sector, as well (Felfe, 2012a, b; Skyt Nielsen et al., 2004; Welteke & Wrohlich, 2019).

The possibility to combine work and family varies between workplaces. There is, in addition, evidence for conformity in childbirth and parental leave decisions at the workplace level (Carlsson & Reshid, 2022; Pink et al., 2014; Welteke & Wrohlich, 2019). Workers either aim for a workplace match in preferences or learn from their peers. There are variations in the childcare leave lengths at different workplaces which are related to workplace characteristics (Österbacka & Räsänen, 2024). Longer leaves generally generate higher costs to workplaces (Ginja et al., 2023), which can have consequences for individual labour market outcomes. However, for instance, Johnsen et al. (2023) show that fathers’ own childcare leave lengths have no impact on their earnings path; instead, it is the leave length in comparison with their peers’ leave length at the workplace level that matters.

There is also support for gender discrimination as a consequence of motherhood if employers see mothers as less competent than non-mothers (de Linde Leonard & Stanley, 2020; Yu & Hara, 2021). A perhaps more obvious explanation for discrimination is that maternity leaves involve expenditure for employers, and consequently, employers are less likely to favour women (Bertrand, 2020; Jessen et al., 2019; Sin et al., 2022). If women are more likely to quit their jobs due to childcare obligations, employers may sort women into jobs with shorter training and less capital, where turnover is less costly. These types of jobs have lower wages in general.

However, uncertainty related to career breaks may also influence employers’ perceptions of their employees. The fertility decision of future mothers is private information and is only revealed when fertility is completed. The final length of the career break(s) is revealed to the employer when the mother returns to the workplace after her childcare leaves and/or decides that her fertility is completed (Barron et al., 1993). Since preferences for childcare may change when giving birth (Kuziemko et al., 2018), the length of childcare leaves might not be a complete certainty even for the mother prior to childbirth. Because of the uncertainty, employers may base their decisions on beliefs. Since women are more likely to take career breaks, it is understandable that women’s labour market outcomes develop slower and are poorer than men’s (Gayle & Golan, 2012). Similarly, Cassidy et al. (2016) argue that asymmetric information about workers’ abilities affects their labour market outcomes. Specifically, if employers are less informed about women’s abilities, they are less likely to promote women.

Asymmetric information on family/work orientation is also likely to affect employers’ decisions on hiring and promoting women. Women can reveal their family/work orientation by their childcare leave length. We can assume that employers’ beliefs regarding the length of childcare leaves are based on previous experience within the workplace. When women return to work, their employers adjust their beliefs. The employer is likely to assume the mother to be family-oriented if she uses longer leaves than the average at the workplace, and work-oriented if she uses shorter.

Another possible explanation for the gender gap is the lack of human capital accumulation or even depreciation during career breaks. The negative impact is likely to vary by leave length, occupation, and sector. Measuring human capital accumulation or the lack of accumulation is tricky, especially regarding workplace-specific human capital. It is possible to deduce the effect of career breaks. For instance, Napari (2010) compares monthly wages among mothers and childless women in the Finnish context and finds only a slight difference. Longer childcare leaves increase motherhood wage penalties somewhat, and he suggests that the lack of human capital accumulation during leave can explain the differences.

The effect of the length of the career break is not necessarily linear. In that sense, the design of family policies is essential. If women are allowed to take childcare leaves and return to their former place of employment, their workplace-specific human capital can be restored quickly after returning to work. Long leaves, on the other hand, are likely to reinforce gender roles and increase the gender gap (Blau & Ehrenberg, 1997; Mincer & Ofek, 1982; Mincer & Polachek, 1974). Maternity rights affect the childcare leave length, and longer leaves have more detrimental effects on mothers’ labour market outcomes, than shorter leaves have (Burgess et al., 2008; Canaan et al., 2022; Hook et al., 2023; Olivetti & Petrongolo, 2017).

### Institutional Background of the Finnish Childcare Leaves

The Finnish family policies belong to the Nordic welfare regime, generally known to be universal and relatively generous and to support two-earner families. However, the Finnish parental leave scheme has characteristics of supporting male-breadwinner families through public support for the home care of children younger than 3 years old.

In the first two decades of the 2000s, before the latest parental leave reform in 2022, the earnings-related parental leave period has been divided into maternity leave (ca 4 months) and parental leave (ca 6 months), which can be divided between mothers and fathers. In addition, fathers have been entitled to paternity leave. The paternity leave length has varied over the years, and a part of it can be used in connection with maternity leave. Mothers generally use the whole parental leave period, while fathers, at most, use the paternity leave. One of the reasons for the slow increase in fathers’ use of paternity leave in the early 2000s was that if fathers extended their paternity leave use beyond the days used in connection with maternity leave (up to 3 weeks), mothers had to transfer a part of their shared parental leave days (Kela, 2020; Saarikallio-Torp & Miettinen, 2021). A paternity quota completely independent of mothers’ parental leave use was introduced as late as 2013. Hence, the low use of paternity leave in the early 2000s hardly influenced the gender pay gap.

The child is around 9 months old when the earnings-related parental leave ends. After that, the parents can choose the dual-earner/dual-career model and enrol their child in publicly subsidised daycare. Municipalities are obliged to offer subsidised daycare to preschool children. Alternatively, parents can choose home care where one of the parents, usually the mother, stays home with their child. In that case, the arrangement resembles the male-breadwinner model. The parent is entitled to a flat-rate home care allowance during home care, with an income-related supplement to low-income families. Some municipalities offer supplements to the flat-rate allowance, henceforth called municipal supplements. Eligibility for the allowance ends when the child turns 3 years old, which coincides with the end of the job-protection period for parents who were employed before childbirth.

The home care allowance was introduced gradually in Finland in 1985 and has since 1990 been available for all children under 3 years of age. It is well-established and is appreciated by parents. Around 90% of families use at least a portion of their home care allowance entitlement. Mothers are most likely to prolong their earnings-related parental leave. However, the length of the home care period varies. Employees must inform their employer at least 2 months before a planned childcare leave by law. For a good cause, the employee may change the time and duration of the leave by notifying the employer at least 1 month before such change is to take effect (ECA 55/2001, 2001).

During our research period, the focal mothers give birth to their first child in 2002–2006. They are followed 5 years prior to and 10 years after their first childbirth. In addition, workplace childcare leave averages are calculated in 1997–2017. Within this research period, only paternal leave benefit regulations changed. However, the policy changes did not increase fathers’ use of shared parental leave (Saarikallio-Torp & Miettinen, 2021). Mothers’ use of home care allowance and the leave lengths have decreased slightly over the years (Haataja & Juutilainen, 2014; Miettinen & Saarikallio-Torp, 2020).

In the early 2000s, the universal flat-rate home care allowance was below 300 Euros per month, while the municipal supplements were between 150 and 200 Euros per month on average. Among first-born children, an increasing share was born in municipalities offering the supplements; between 2002 and 2006, the share increased from around 45 to 55% (Kela, 2018; Räsänen et al., 2019). In comparison, the average cost for public daycare was around 190 Euros per child per month, and the maximum fees were 220 Euros for the first and 190 Euros for the second child. Hence, parents with children aged 1 or 2 can choose whether one of them—the mother—takes care of the child or both parents work and use subsidised daycare.^1^

The Finnish childcare leave policies allow mothers to choose the length of their childcare leave, but with varying benefit levels. Family-oriented mothers are likely to choose more extended and several home care periods, while work-oriented mothers are likely to return to work faster and use fewer home care periods. For instance Gruber et al. (2023) find large short-run child penalties in Finland and show that they are almost completely due to the home care allowance.

The individual choice of childcare leave length is likely to reveal family or work orientation. Employers may believe that family orientation is correlated with reduced productive capability, at least temporarily. In addition, a lack of childcare support can be interpreted as a disadvantage when hiring mothers (Bedi et al., 2022). However, all children have access to publicly subsidised daycare in Finland. Finnish mothers can choose between subsidised home care and public daycare until the child turns 3. Neither employers nor employees have to worry about access to daycare. We proceed to describe the Finnish data.

## Data

### Sample

Our data are a 70% simple random sample of all mothers giving birth between 1997 and 2017. The data set stems from register data compiled by Statistics Finland and the Social Insurance Institution of Finland. Household information is added; hence, information on fathers is also available. We limit the sample to new parents in the time period 2002–2006 who are employed the year prior to birth with monthly earnings information available in the data, i.e. self-employed are not included. The parents are followed 5 years before and 10 years after the birth of their first child. We observe the number of children 10 years after their first birth, which gives us information on cumulative fertility. We extend the data with background information on education, marital status, and workplace characteristics from linked employer–employee data for all mothers and fathers.

We use monthly earnings from the Structure of Earnings Statistics (SES) in the analyses. The SES is somewhat restricted. Overall, the private sector coverage is sufficient, and we have full coverage of the public sector. The advantage of the SES is that it offers precise measures of compensation from work on a monthly level.^2^

Most mothers use all maternity and shared parental leave benefits, while a small fraction of fathers (under 2%) uses shared parental leave (Saarikallio-Torp & Miettinen, 2021). For our purposes, there is insufficient variation in parental leave length among mothers. However, childcare leaves that continue after parental leave vary considerably between mothers; some mothers return to work immediately after the parental leave ends when the child is around 9 months old, while some return when the job-protection period ends when the child is 3 years old. On average, mothers who were employed pre-birth return to work before the child turns 2 years old, while mothers out of the labour market pre-birth stay home longer (Räsänen et al., 2019).

We use register data on monthly home care allowance payments to the mother as information on the length of childcare leaves. Following the birth of the first child, we calculate cumulative childcare leaves annually for all mothers in the sample up to 10 years after their first birth.

We exploit the employer–employee link to calculate average childcare leaves for all workplaces from 1997 to 2017. First, we calculate the leave duration for all female employees. Second, we calculate the yearly average workplace-specific cumulative childcare leaves. We calculate average childcare leaves for all workplaces where at least three women have previous childcare leave histories, excluding the sample mothers’ childcare leave lengths at their respective workplace.^3^

**Table 1:** Descriptive statistics for the sample of mothers and fathers $t=-1$, $t=0$, or $t=10$ years relative to first childbirth

	Mothers	Fathers
Annual earnings ($t-1$)	28,986.46	37,973.62
Monthly earnings ($t-1$)	2,357.56	3,015.86
Education, basic ($t-1$)	0.05	0.10
Education, secondary ($t-1$)	0.34	0.46
Education, tertiary ($t-1$)	0.61	0.44
Age ($t=0$)	29.26	31.49
Employment ($t+10$)	0.86	0.85
Annual earnings ($t+10$)	32,526.91	49,133.27
Monthly earnings ($t+10$)	3100.30	4147.01
Avg. number of children ($t+10$)	2.28	2.22
N	44,708	40,328

Childcare leave after parental leave ($t+10$)	Mean	Sd
One child	11.22	9.47
Two children	20.17	14.28
Three children	31.22	19.02
Four or more children	41.17	22.69
All	23.06	17.89

Earnings are in 2017 values by CPI and comprise all wages, salaries, and fringe benefits related to compensation for work. Average childcare leaves exclude earnings-related parental leave (around nine to ten months per child). Monthly earnings are conditional on employment and included in the Structure of Earnings Statistics (SES). Monthly earnings information is available for approximately 2/3 of the employed

Table 1 describes the sample. The mothers are on average 29 years old when they have their first child (at $t=0$). They are well educated, with 34% having secondary education and 61% having tertiary education. Their average monthly earnings pre-birth are below 2400 Euros. The mothers in this sample have had 2.28 children on average 10 years after their first birth, which is somewhat higher than the total fertility rate, in the 2000s, which has ranged between 1.72 and 1.87. Considering that the sample includes mothers only, the higher average birth rate is not surprising and is also in line with completed fertility rates among Finnish mothers in the same age range (Jalovaara et al., 2022).

The variable of interest, the childcare leave, is presented as the cumulative childcare leaves 10 years after the first birth, which points out that mothers have stayed home, on average, for almost 2 years with their child(ren) in addition to their earnings-related parental leave period(s). However, some use home care allowance for a considerably longer, while others for a considerably shorter time. The variation is partly due to the variation in number of children and partly due to variation in leave lengths. Mothers with one child use less than a year on average, while mothers with two children use 20 months on average. Higher parity mothers use on average 10–11 additional months per child.

The fathers in the sample are on average 2 years older than the mothers when having their first child. They are not as well educated as mothers, 46% having secondary education while 44% having tertiary education. Their average monthly earnings pre-birth are around 3000 Euros.

When comparing the earnings before the first birth and 10 years after, the earnings trend is positive. Mothers have on average 32% higher monthly earnings, and the corresponding increase for fathers is 38%.

## Empirical Strategy

### Event Study

We follow Kleven et al. (2019b) in estimating the impact of childcare leaves on mothers’ labour market outcomes by using an event study approach. They argue that the method identifies both short- and long-run child penalties. Recent studies by e.g. Kleven et al. (2019a), Kleven et al. (2019b) and Sieppi and Pehkonen (2019) estimate average child penalties for women in several labour market outcomes. The method makes it possible to study the effects of variations in childcare leave lengths. However, to receive a causal interpretation of the impact of variations in leave lengths, exogenous variations are required.^4^

We compare mothers’ yearly cumulative childcare leave lengths to their workplace cumulative averages annually over a 10-year period. The cumulative childcare leave length adds information on leave length for the first child and possible subsequent children. By relating to workplace averages, we are able to take workplace norms and specifically consequences of leave length in relation to workplace averages into account.

Workers at the same workplace and within the same occupation are more homogeneous than individuals drawn from a random sample, and women conform to workplace norms regarding childbirth and childcare leaves. The length of childcare leave among mothers is associated with employment sector, number of employees, peers’ leave length, and the share of women in the workplace. By relating to workplace averages, we can mitigate the fact that individual characteristics do not pick up typical workplace characteristics, which reduces possible omitted variable bias (Carlsson & Reshid, 2022; Frederiksen, 2008; Korkeamäki & Kyyrä, 2006; Pehkonen et al., 2017; Pink et al., 2014; Wilner, 2016; Österbacka & Räsänen, 2024). In addition, deviations from the workplace average are likely to affect career developments post-birth.

Earnings-related parental leave ends when the child is around 9 months old, and employers do not expect mothers to return to work before the parental leave period ends. Mothers can demonstrate their family/work orientation (and differentiate from their peers) by the timing of their return after their parental leave period ends.

We modify the event study approach for the average effects in Kleven et al. (2019b) and contrast the average effects by childcare leave length and estimate for fathers, *m*,

1 $$\begin{aligned} Y^m_{iyt} = \sum _{j \ne -1} \alpha ^m_j \hbox{event}_{tj} + \beta ^m_X X^m_i + \delta ^m_{a} + v^m_{t} + u^m_{mn} + \varepsilon ^m_{iyt}, \end{aligned}$$

and for mothers, *w*,

2 $$\begin{aligned} Y^w_{iyt}&= \sum _{j=-5}^{-2} \alpha ^w_j \hbox{event}_{tj}  \\&\quad +\sum _{j \ge 0} \gamma ^w_j \left( \hbox{event}_{tj} \times \hbox{longer}_{tj}=0\right) + \sum _{j \ge 0} \eta ^w_j \left( \hbox{event}_{tj} \times \hbox{longer}_{tj}=1\right)  \\&\quad + \beta ^w_X X^w_i + \delta ^w_{a} + v^w_{y} + u^w_{mn} + \varepsilon ^w_{iyt}, \end{aligned}$$

where the monthly earnings $Y^p_{iyt}$ for parent *i*, (parent, $p =m,w$) in calendar year *y*, are measured *t* years relative to childbirth. In the model, $\delta ^p_a$ is the age fixed effect, $v^p_y$ is the year fixed effect, $u^p_{mn}$ is the municipal fixed effect, $\varepsilon ^p_{iyt}$ is the error term, and the matrix $X^p_i$ includes education pre-birth. The errors are clustered by age, year, and municipality.

By relating to the year prior to birth ($j=-1$), the dummy variable $\hbox{event}_{tj}$ equals one when the year relative to childbirth ($t=j$), 5 to 2 years prior to or up to 10 years after. The coefficients $\alpha ^m_j$ measure the effects among fathers. The corresponding coefficients for mothers, $\alpha ^w_j$, measure the effects pre-birth, and $\gamma ^w_j$ measure the effects when her cumulative childcare is shorter or equally long as the workplace average post-birth the corresponding year ($\hbox{longer}_{tj}=0$). In comparison, $\eta ^w_j$ measure the effects when her cumulative childcare is longer than the workplace average post-birth the corresponding year ($\hbox{longer}_{tj}=1$). The coefficients $\gamma ^w_j$ and $\eta ^w_j$ measure the outcomes of taking shorter, respectively, longer childcare leaves than the average at the workplace.

The level effects can be converted into percentages, $\hbox{perc}_t$, where the average time trends are related to the corresponding predicted counterfactual outcome in the absence of childbirth, e.g. for mothers, ${\tilde{Y}}^w_{iyt}$. Hence, the average earnings trend for mothers, *w*, can be calculated over the years as $\hbox{perc}^w_{t} = ({\hat{\alpha }}^w_t + {\hat{\gamma }}^w_t + {\hat{\eta }}^w_t)/{\textbf{E}}[{\tilde{Y}}^w_{iyt}|t]$, and for fathers, *m*, and as $\hbox{perc}^m_{t} = ({\hat{\alpha }}^m_t) /{\textbf{E}}[{\tilde{Y}}^m_{iyt}|t]$.

### Instrument

Longer or shorter than workplace average childcare leaves may be endogenous since unobservable individual characteristics, such as family/work preferences or post-childbirth selection, may affect leave duration. In addition, women conform to their peers. Municipal supplements to home care allowance create exogenous variations in leave lengths. Higher home care allowances increase the length of childcare leaves (Kosonen, 2014; Österbacka & Räsänen, 2022).

We instrument leave length by an indication of whether the mother’s home municipality offered a supplement when the child is born, $Z_{t = 0}$.^5^ We follow the robustness check by Kleven et al. (2019b) and interact the second row in Eq. 2 by $Z_{t=0}$. By interacting the exogenous instrument with the event study specification, instead of using a more traditional IV methodology, we get full dynamic effects after childbirth and employment re-entry.

A local average treatment effect (LATE) is a meaningful interpretation of the IV results where municipal supplements’ binary treatment variable affects a group of compliers. In our context, compliers react to the instrument turning on ($Z_{t=0}=1$) and off ($Z_{t=0}=0$). Family-oriented mothers who use longer childcare leaves regardless of the instrument’s value are always-takers. In contrast, work-oriented mothers who use shorter childcare leaves regardless of the instrument’s value are never-takers. The coefficients $\gamma ^w_j$ in Eq. 2 representing mothers who use equally long or shorter childcare leaves than their workplace averages are split into two groups depending on the value of $Z_{t=0}$; if $Z_{t=0}=1$, the coefficients represent never-takers and if $Z_{t=0}=0$, they represent compliers and never-takers. Similarly, $\eta ^w_j$ representing mothers who use longer childcare leaves than their workplace averages are split into two groups depending on the value of $Z_{t=0}$; compliers and always-takers if $Z_{t=0}=1$ and always-takers if $Z_{t=0}=0$. These groups are illustrated in Table 2.

**Table 2:** The categories of never-takers, compliers, and always-takers identified by the instrument, municipal supplement at $t=0$, $Z_{t=0}$, and leave length in relation to their workplace average childcare leave among peers

	Childcare leave compared to peers	
	Shorter ($\gamma _j^w$ in Eq. 2)	Longer ($\eta _j^w$ in Eq. 2)
Instrument off, $Z_{t=0}=0$	Compliers and never-takers	Always-takers
Instrument on, $Z_{t=0}=1$	Never-takers	Compliers and always-takers

The difference between the outcomes for the two groups of those represented by $\gamma ^w_j$ and $\eta ^w_j$ in Eq. 2 gives more explicit indications of the impact of self-selection. Never-takers and always-takers do not conform to their peers nor to the supplement level. Instead, they commit to work or family, respectively, i.e. self-select. The groups, including compliers, do not necessarily use shorter or longer cumulative childcare leaves than their workplace averages because of self-selection; instead, they comply with the supplement level. Or in other words, they are affected by the level of the municipal supplement.

Some municipalities provide supplements during the whole sample period (first births in the period 2002–2006), while other municipalities do not offer any supplements. Some municipalities switch their treatment status from not offering to offering supplements. Municipalities switching their treatment type provide most of the variation to identify the LATE IV estimate. All the different categories of municipalities comprise different sizes as well as degree of urbanisation and rurality.

## Results

### The Effect of Childcare Leave Length on Child Penalty

**Fig. 1:**
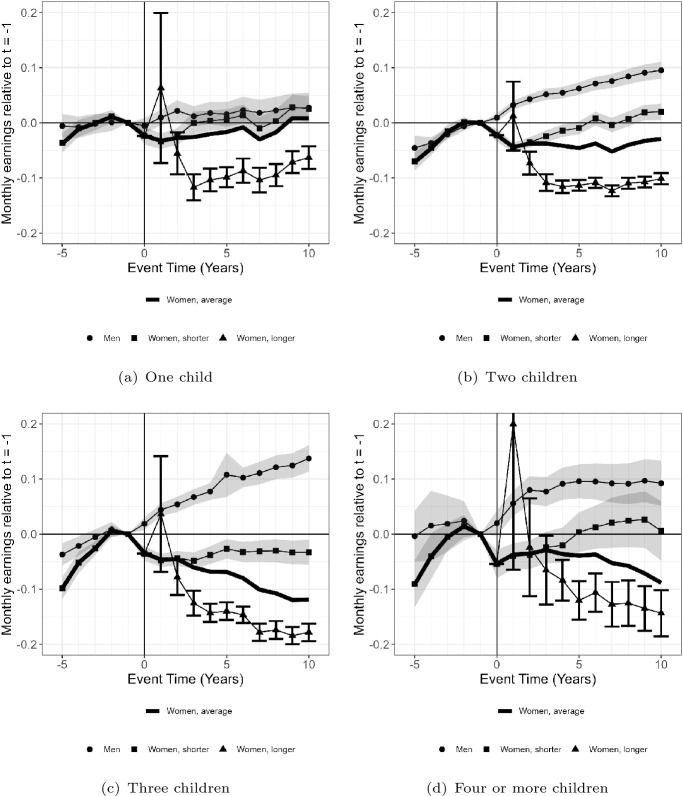
Monthly earnings, ranging between 5 years prior to and 10 years after the first birth, related to monthly earnings pre-birth $(t-1)$ among fathers and mothers by parity. *Note*: The event time results are calculated as the percentage of the counterfactual outcome at time $t - 1$: $\hbox{perc}^m_{t} = ({\hat{\alpha }}^m_t) /{\textbf{E}}[{\tilde{Y}}^m_{iyt}|t]$ (from equation 1) for men and $perc^w_{t} = ({\hat{\alpha }}^w_t + {\hat{\gamma }}^w_j + {\hat{\eta }}^w_j)/{\textbf{E}}[{\tilde{Y}}^w_{iyt}|t]$ (from Eq. 2) for women. The line with dots represents fathers; the black line represents average mothers; the line with squares represents mothers with equally long or shorter childcare leaves than their workplace averages; the line with triangles represents mothers with longer childcare leaves than their workplace averages.

Figure 1 presents the event study estimates in monthly earnings related to the pre-birth level as percentages by parity. The odd increase in the first year after birth among mothers using longer childcare leaves than their peers is due to a low number of observations and unusual individuals working in unusual workplaces, i.e. returning within a year after childbirth but using longer childcare leaves than their peers. The earnings trends among one-child parents remain flat post-birth. The exception is among mothers who use longer childcare leaves than their peers; they experience a clear penalty. Two-child parents represent the largest group of parents in the sample and their earnings trends diverge. After their first birth, fathers’ monthly earnings trend increases. Mothers using equally long or shorter childcare leaves than their workplace averages experience a small drop in monthly earnings post-birth, but their earnings trend recovers within 5 years after their first birth. Mothers who use longer childcare leaves than the average at their workplace, on the other hand, experience a clear drop in monthly earnings, and their earnings do not recover.

The earnings trends among three-child parents and parents with four or more children show similar patterns. The earnings trend among fathers evolves faster, while the earnings trend among mothers prolonging their childcare leave is around 15–20% lower 10 years after their first birth. The number of families with four or more children in the sample is low, and the standard errors for the estimated trends are large.

**Fig. 2:**
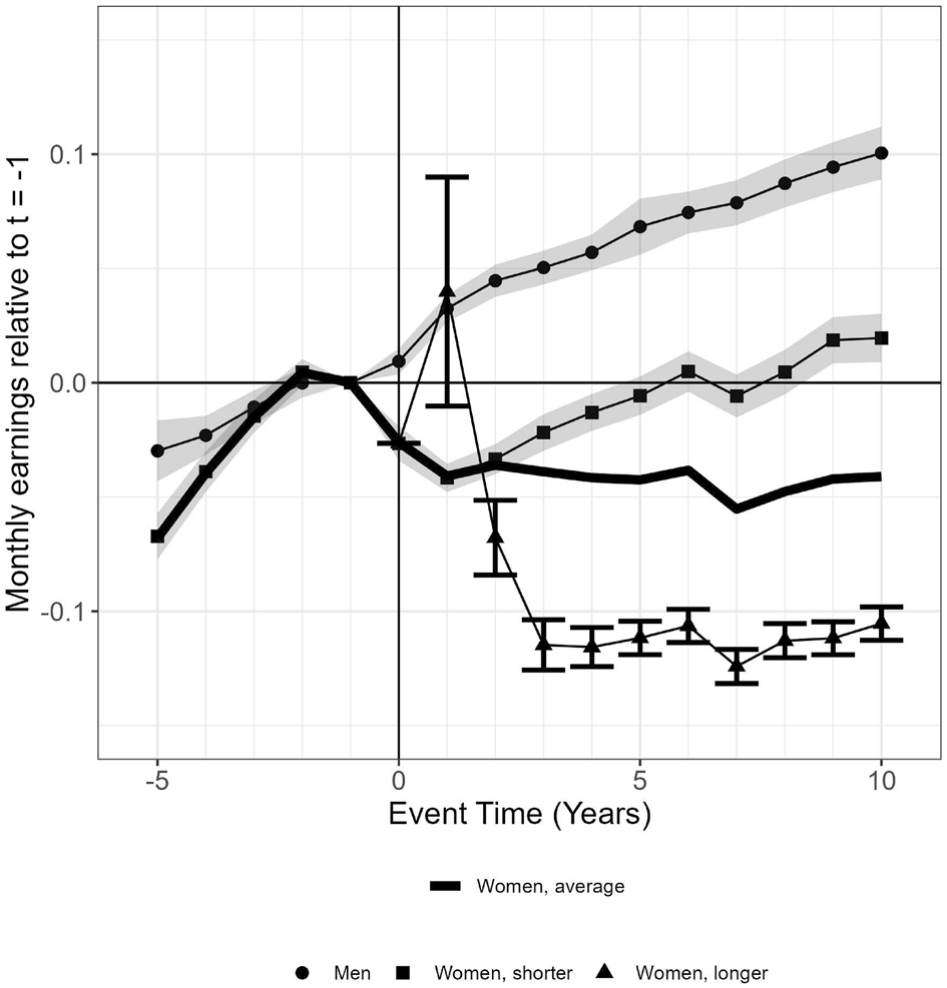
Monthly earnings, ranging between 5 years prior to and 10 years after the first birth, related to monthly earnings pre-birth $(t-1)$ among fathers and mothers. *Note*: The event time results are calculated as the percentage of the counterfactual outcome at time $t - 1$: $\hbox{perc}^m_{t} = ({\hat{\alpha }}^m_t) /{\textbf{E}}[{\tilde{Y}}^m_{iyt}|t]$ (from Eq. 1) for men and $\hbox{perc}^w_{t} = ({\hat{\alpha }}^w_t + {\hat{\gamma }}^w_j + {\hat{\eta }}^w_j) /{\textbf{E}}[{\tilde{Y}}^w_{iyt}|t]$ (from Eq. 2) for women. The line with dots represents fathers; the black line represents average mothers; the line with squares represents mothers with equally long or shorter childcare leaves than their workplace averages; the line with triangles represents mothers with longer childcare leaves than their workplace averages.

Figure 2 presents the overall results. On average, fathers’ monthly earnings increase after their first birth. Mothers experience, on average, a drop in monthly earnings post-birth, and their earnings trend is almost 5% lower 10 years after their first birth. Mothers who use equally long or shorter childcare leaves than their workplace averages experience a clear drop in monthly earnings post-birth, but after their first child is 1 year old, their earnings start to recover, and they pick up their pre-birth level when their first born starts school. Their long-run child penalty is 10%. Mothers who use longer childcare leaves than their workplace average, on the other hand, experience a clear drop in monthly earnings, and their earnings do not recover. Ten years after their first birth, their monthly earnings are more than 10% lower than pre-birth, when age, year, and municipal fixed effects, as well as education pre-birth, are controlled for. Their long-run child penalty is 22%.^6^ Two-child parents represent the largest group of parents in the sample, and their earnings trends are similar to the trends among all parents.

Women self-select the number of children and the consecutive childcare leave lengths. However, they tend to conform to the workplace norm or they change to jobs more in congruence with their own norms. The average effects of equally long and shorter childcare leaves, or correspondingly longer than the workplace averages, have noticeable effects on the earnings trends. At this point, however, it is impossible to disentangle the causes for these relationships.

### Instrument and Childcare Leave Length

We proceed with examining the impact of the instrument for all mothers. Table 3 presents descriptive statistics for the different groups identified by the instrument, municipal supplement at childbirth for all mothers. Ten years after the first childbirth, 26% of the mothers are categorised as never-takers, while 22% as always-takers. Among mothers that are likely to react to the supplement level, 32% give birth in a municipality without supplement and use shorter cumulative childcare leaves than their peers, while 20% give birth in a municipality with supplement and use longer cumulative childcare leaves than their peers.^7^

**Table 3:** Descriptive statistics for the groups identified by the instrument, municipal supplement at $t=0$

Childcare leave compared to peers	Shorter		Longer	
	$Z = 1$	$Z = 0$	$Z = 1$	$Z = 0$
	Never-takers	Compliers and never-takers	Compliers and always-takers	Always-takers
Annual earnings ($t-1$)	33,721.19	29,049.58	30,856.14	26,858.88
Monthly earnings ($t-1$)	2718.91	2346.31	2503.69	2186.98
Part-time ($t-1$)	0.08	0.12	0.10	0.15
Education: basic ($t-1$)	0.04	0.03	0.04	0.04
Secondary ($t-1$)	0.25	0.32	0.28	0.36
Tertiary ($t-1$)	0.71	0.64	0.68	0.60
Age ($t=0$)	30.50	29.60	29.67	28.92
Avg. number of children ($t+10$)	1.93	1.98	2.41	2.47
Annual earnings ($t+10$)	45,753.28	38,448.99	37,027.66	32,130.93
Monthly earnings ($t+10$)	3593.81	3066.80	3030.29	2635.07
Part-time ($t+10$)	0.13	0.16	0.27	0.29
Childcare leave, mean ($t+10$)	10.51	9.71	35.53	37.17
Childcare leave, peers ($t+10$)	21.84	22.84	21.57	22.37
Difference to peers ($t+10$)	$-$ 11.33	$-$ 13.13	13.96	14.80
Difference to peers, SD	7.07	7.50	11.40	11.94
N	7949	9982	6217	6778
Share of observations ($t+10$)	25.7	32.3	20.1	21.9

Since an individual can change groups over the years, the descriptive statistics are presented given that an individual belongs to the corresponding group at $t=10$ and has positive monthly earnings

Mothers living in a municipality that offers the supplement at $t=0$ have a higher education and earnings pre-birth. They also work part time to a somewhat lower extent and are around a year older when giving birth.

Mothers who choose shorter cumulative childcare leaves than their workplace averages seem to choose somewhat different careers pre-birth than mothers choosing longer childcare leaves, which indicates signs of family/work orientation already pre-birth. Earnings and education are somewhat higher among mothers using shorter cumulative childcare leaves. They have somewhat below 2 children on average and use less than a year in childcare on average in addition to their earnings-related parental leave. Mothers using longer cumulative childcare leaves have almost 2.5 children on average and use around 3 years in childcare. Peers spend almost 2 years on cumulative childcare leave in addition to their earnings-related parental leave irrespective of to which group the sample individual is categorised.

The labour market outcome differences aggravate 10 years after the first birth. Mothers using shorter cumulative childcare leaves than their workplace averages experience around a 30% increase in monthly earnings on average. Yearly earnings increase somewhat more. The corresponding increases among mothers using cumulative longer childcare leaves than their workplace averages are around 20%. The differences in part-time work at $t+10$ are also noticeable. However, the labour market outcome differences are hardly due to the differences in number of children since that difference is comparatively small.

In the event study estimates, we control for education pre-birth in addition to age, year, and municipal fixed effects, which take deviations between groups pre-birth into account. The predicted earnings trends are also related to pre-birth earnings and counterfactual outcomes where the impact of childbirth is omitted, which also considers pre-birth choices.

**Fig. 3:**
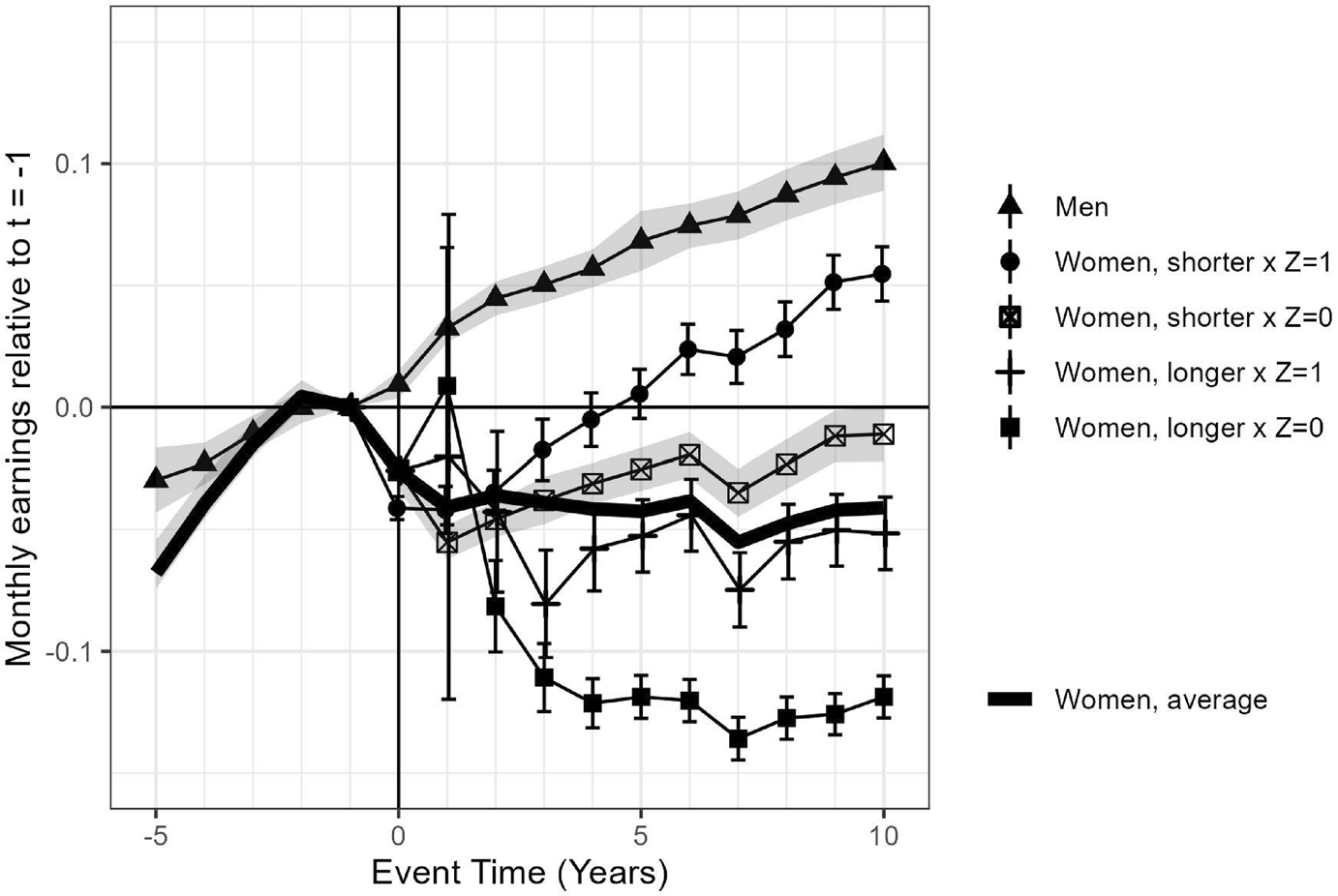
Monthly earnings, ranging between 5 years prior to and 10 years after the first birth, related to monthly earnings pre-birth $(t-1)$ among fathers and mothers, where post-birth events among mothers are interacted with an instrument, $Z_{t=0}$. *Note*: The instrument, $Z_{t=0}$, indicates if the mother’s home municipality offers a home care allowance supplement when the first child is born. The line with triangles represents fathers; the black line represents average mothers; the line with dots represents mothers with equally long or shorter childcare leaves than their workplace averages with municipal supplements (never-takers); the line with crosses represents mothers with equally long or shorter childcare leaves than their workplace averages without municipal supplements (compliers and never-takers); the line with dashes represents mothers with longer childcare leaves than their workplace averages with municipal supplements (compliers and always-takers); the line with squares represents mothers with longer childcare leaves than their workplace averages without municipal supplements (always-takers).

In Fig. 3, the estimates when adding the instrument are presented. We can assume that the compliers do not necessarily make their choices based on workplace norms but comply with the municipal supplement. Both groups experience a child penalty. However, the earnings trend of mothers using shorter than the workplace average in municipalities not offering any supplement recovers almost fully to their pre-birth level 10 years after first birth. The earnings trend of mothers using longer than the workplace average in municipalities offering a supplement, on the other hand, falls behind their pre-birth level and their earnings trend does not recover. The difference between the two groups of compliers, affected by the municipal supplement difference, results in a long-run child penalty of 13% and 17%, respectively. The difference between these groups is the LATE IV estimate of the municipal supplement. A higher allowance level extends the childcare leave and aggravates the long-run child penalty by around 4 percentage points.

Never-takers do not conform to their peers nor comply with the higher supplement levels, and they experience a clear drop in monthly earnings after childbirth, but their earnings trend recovers over the years. Ten years after their first birth, there is a comparatively small gap in comparison with the trend among fathers. Their corresponding long-run child penalty is only around 6%. This group self-selects into shorter childcare leaves than their workplace averages and reveals work orientation, which is rewarded.

Always-takers do not conform to their peers nor comply with the non-existent supplement, and their monthly earnings drop remarkably. Their earnings trend does not recover but remains around 12% below the pre-birth level, which corresponds to a long-run child penalty of 24%. This group self-selects into longer childcare leaves than their workplace averages and reveals family orientation, which is penalised.

In Appendix Fig. 7, the results are presented by parity. The results correspond to the overall results presented in Fig. 3. The number of observations is comparatively low in one child and four or more children families. The estimated earnings trends overlap in these families, but always-takers seem to lag behind. However, it is worth noticing that the relevance of the instrument is weak for parity of one and four or more children. Most families have two or three children, for which the instrument has relevance.^8^ Most families have two, and to a lesser extent three, children. Among these families, there are clear differences. The earnings trends of never-takers recover, while always-takers are penalised. The two groups of compliers are penalised, and the long-run difference due to different leave lengths between the groups is around 5 percentage points.

A relevant concern about the results is that the different groups of mothers self-select into different careers pre-birth and that this self-selection explains the earnings trends post-birth. If that were the case, work-oriented mothers would self-select into more prestigious careers, while family-oriented mothers would self-select into careers with fewer prospects already pre-birth. The means presented in Table 3 indicate that the labour market outcomes among groups of mothers do, indeed, differ pre-birth. Work-oriented never-takers are more highly educated and have higher earnings pre-birth than family-oriented always-takers. However, mothers living in municipalities offering the supplement are more highly educated and have higher earnings irrespective of being a never-taker or a complier, while the opposite holds for mothers living in a municipality not offering the supplement. We control for education pre-birth and include municipal fixed effects to control for these differences.

Another concern is that a possible explanation for more considerable penalties among mothers using longer leaves is the lack of human capital accumulation or depreciation in addition to self-selection. Our results show that mothers using shorter childcare leaves than their workplace averages experience a considerably lower penalty than mothers using longer childcare leaves than their workplace averages, which is consistent with human capital depreciation or lack of accumulation due to longer leaves. On the other hand, the two groups of mothers staying home with their children shorter, respectively, longer than their workplace averages but distinguished by the instrument should have similar outcomes, which they do not have. Always-takers have higher penalties than compliers even though they use on average approximately equally long childcare leaves. Similarly, never-takers have lower penalties than compliers even if they use on average approximately equally long childcare leaves.

The two groups using shorter and longer childcare leaves than their workplace averages differ somewhat pre-birth. In our analyses, we control for education pre-birth and include age, year, and municipal fixed effects, and the outcomes post-birth differ due to the instrument. The differences in outcomes cannot be explained by human capital depreciation only. Instead, self-selection and how employers react to the self-selection are more likely candidates for causing the differences in outcomes between the two groups of mothers using longer and shorter childcare leaves than their workplace average, respectively. Employers may discriminate mothers. However, they may also react to work/family orientation mothers reveal by self-selecting into shorter or longer leaves than the workplace average.

### Identification Assumptions

**Fig. 4:**
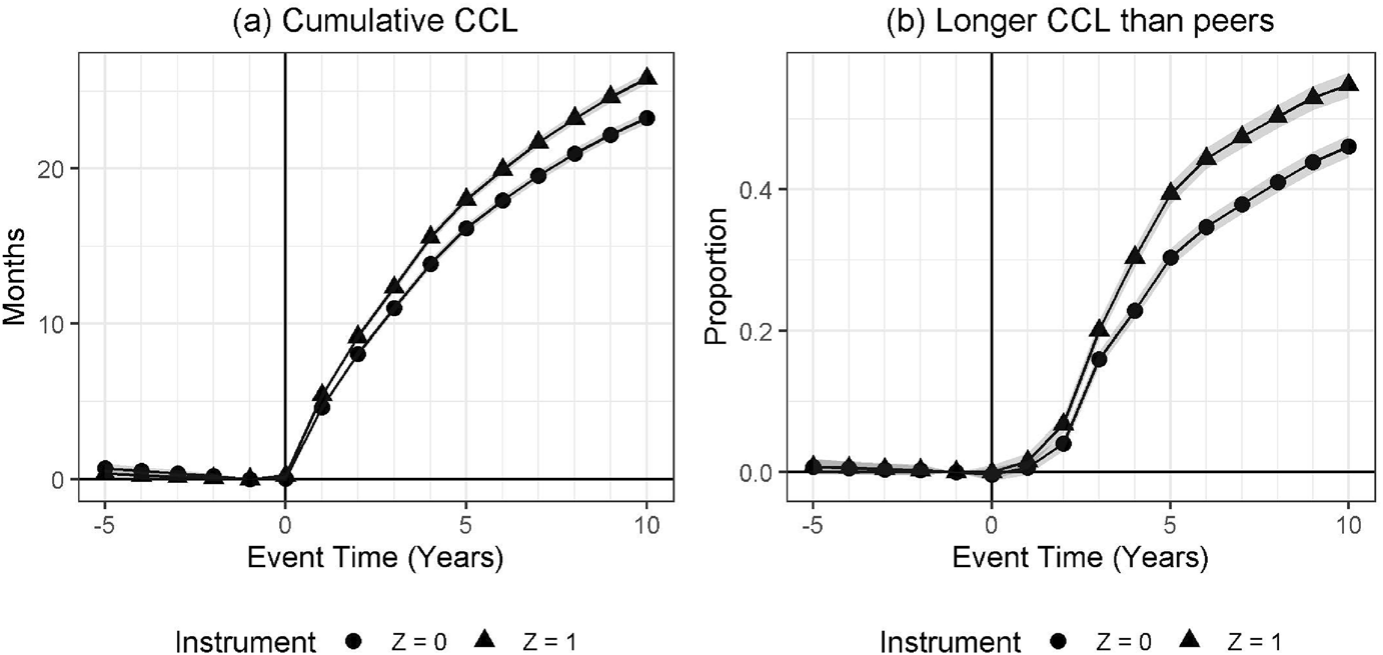
First stage of the instrument, $Z_0$, the impact of a municipal home care allowance supplement at childbirth. *Note*: In both panels, the outcomes are estimated separately for a sample where $Z_{t=0} = 0$ and $Z_{t=0} = 1$, respectively. The panels show event time estimates compared to $t - 1$ of (**a**) cumulative childcare leave length (all women); (**b**) the proportion of mothers with longer childcare leaves than average peers (all employed).

Figure 4 presents results of the relevance of the instrument. In both cases, the pre-trends are overlapping. Panel (a) shows that when a municipality offers a supplement, mothers prolong their cumulative childcare leave. In addition, panel (b) shows that mothers eligible for a municipal supplement are more likely to take longer than workplace average childcare leaves than non-eligible mothers. The deviation from the workplace averages can be attributed to differences in timing of the introduction, or increase, of the municipal supplement. Mothers giving birth earlier are not necessarily exposed to the supplement.

Independence assumption and exclusion restriction require that the instrument, home care allowance supplement at t = 0, is exogenous to family characteristics and affects labour market outcomes only through longer childcare leaves. Municipalities increasing the supplement in response to higher female employment and higher demand for public childcare may violate the independence assumption. Table 3 shows that the average number of children 10 years after the first birth is not higher in municipalities offering the supplement the year at first birth. Less than a third of municipalities considered the home care supplement to reduce demand for public daycare in 2016, which implies that other reasons than a high demand for public daycare are more critical for the introduction of supplements (Lahtinen & Selkee, 2016). Furthermore, municipalities are obliged, by law, to offer daycare to children below school age.

Municipal decision-making delays introducing new supplements since the municipal council decides on the level of the supplements. New municipal councils are elected every fourth year, further complicating the decision-making. Several reasons unrelated to the labour market can cause the council to increase or reduce the supplement. For example, a desire to offer services to families or home care is considered better for children, which are reasons for municipalities to supplement home care (Miettunen, 2008; Repo et al., 2019). Overall, institutional settings, namely municipal decision-making and elections, ensure that the decision to change the supplements is delayed and is exogenous to the families.

Labour market legislation and eligibility criteria for the home care supplement ensure that the control group is untreated, and the instrument affects only the treatment group. Crossover bias is unlikely for several reasons. First, only residents are eligible for the supplement and all families, regardless of their employment status, are eligible. Second, workplace-level labour market regulations are set at the state level, not at the municipal level. Lastly, families may move to gain higher supplements, which would mean that families could manipulate the inclusion into treatment. However, few families move from non-supplement municipalities to municipalities offering supplements when children are young. Between 1 year before the first childbirth and the year when the child is 2 years old, 2.7% of women have moved from a municipality that did not offer to a municipality offering supplements. In comparison, 22.6% of women moved to a new municipality between pregnancy when the first child turned 2 years old.

Appendix Figure 5 shows that the instrument does not affect fertility nor the average length of childcare leave among peers. Mothers and their peers show similar trends irrespective of the value of the instrument, which supports the exclusion restriction. In addition, Appendix Fig. 6 shows that the instrument does not significantly impact the probability of positive earnings or worked monthly hours. However, mothers living in a municipality offering a home care allowance supplement when their first child is born have lower monthly and annual earnings post-birth. The pre-trends for the different groups of mothers identified by shorter or longer childcare leaves than their peers and the instrument are similar. Furthermore, institutional settings arguably provide the instrument not to affect the outcome through unobservable characteristics included in the error term.

**Fig. 8:**
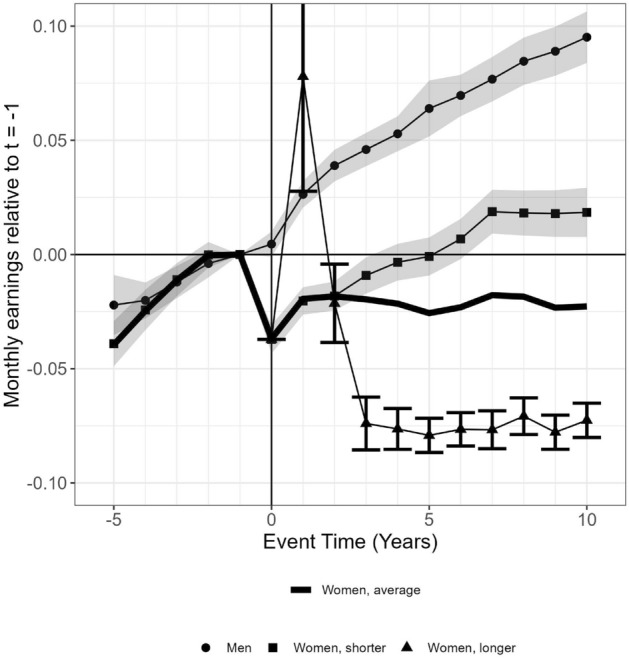
Monthly earnings, ranging between 5 years prior to and 10 years after the first birth, related to monthly earnings pre-birth $(t-1)$ among fathers and mothers working full time. *Note*: The event time results are calculated as the percentage of the counterfactual outcome at time $t - 1$: $\hbox{perc}^m_{t} = ({\hat{\alpha }}^m_t) /{\textbf{E}}[{\tilde{Y}}^m_{iyt}|t]$ (from Eq. 1) for men and $\hbox{perc}^w_{t} = ({\hat{\alpha }}^w_t + {\hat{\gamma }}^w_j + {\hat{\eta }}^w_j) /{\textbf{E}}[{\tilde{Y}}^w_{iyt}|t]$ (from Eq. 2) for women. The line with dots represents fathers; the black line represents average mothers; the line with squares represents mothers with equally long or shorter childcare leaves than their workplace averages; the line with triangles represents mothers with longer childcare leaves than their workplace averages.

**Fig. 9:**
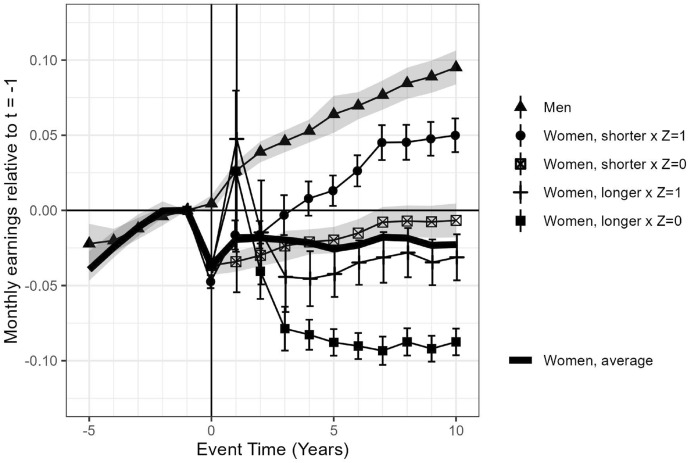
Monthly earnings, ranging between 5 years prior to and 10 years after the first birth, related to monthly earnings pre-birth $(t-1)$ among fathers and mothers, where post-birth events among mothers are interacted with an instrument, $Z_{t=0}$. Parents working full time. *Note*: The instrument, $Z_{t=0}$, indicates if the mother’s home municipality offers a home care allowance supplement when the first child is born. The line with triangles represents fathers; the black line represents average mothers; the line with dots represents mothers with equally long or shorter childcare leaves than their workplace averages with municipal supplements (never-takers); the line with crosses represents mothers with equally long or shorter childcare leaves than their workplace averages without municipal supplements (compliers and never-takers); the line with dashes represents mothers with longer childcare leaves than their workplace averages with municipal supplements (compliers and always-takers); the line with squares represents mothers with longer childcare leaves than their workplace averages without municipal supplements (always-takers).

Lastly, higher home care allowances may delay employment re-entry and mothers who return later than their peers may work part time, while their peers work full time which would aggravate the estimates. We test whether the difference between full-time and part-time work can explain part of the observed child penalty. Figures 8 and 9 in Appendix present the earnings trends for parents working full time. These results indicate that part-time work explains a part of the child penalty. However, Figs. 8 and 9 are nearly similar to Figs. 2 and 3, including all mothers. The earnings trends for men are almost identical. The earnings trends for mothers working full time and with shorter leaves than their peers are similar, but recover somewhat faster than the whole sample including mothers working part time. The earnings trends among mothers working full time with longer leaves than their peers recover even faster compared to the sample including mothers working part time. Part-time work after child birth slows down the earnings trend, especially among mothers taking longer leaves than their peers. However, the patterns are similar, the contribution of part-time work to the long-run child penalty is small, and it does not alter the effect of the instrument.

## Conclusions

The child penalty in pay is long-lasting in Finland. The penalty is lower among mothers participating in the labour market than the overall average but heterogeneous. In this paper, we exclude non-working individuals from the studied sample. In addition, a person needs to be included in the Structure of Earnings Statistics (SES), which makes attrition a limitation of our study. However, our sample includes parents who were employed before childbirth and they are more likely to be included in the sample in the long run. For instance, 9 out of 10 parents remain employed in the long run, and 7 out of 10 are included in the SES 10 years after their first childbirth.

Another limitation to our study is that longer leaves are not necessarily manifested preferences. Longer leaves can for instance be associated with unforeseen health-related reasons for the child and/or mother. Since we focus on working mothers, we may not include the most severely disadvantaged.

We find that mothers using equally long or shorter childcare leaves than their workplace average have a 10% long-run earnings penalty, while mothers using longer childcare leaves have a 22% earnings penalty.^9^ The possibility to choose leave lengths up to 3 years in the Finnish context is of importance for the estimate child penalties. Some mothers self-select into longer or shorter childcare leaves, while others comply with varying home care allowance levels. We argue that the variation in childcare leave lengths contributes to different labour market outcomes and the child penalty among working mothers.

Family-oriented mothers are likely to have more children and take care of them for longer periods. Work-oriented mothers, on the other hand, are likely to have fewer children and return to work faster. To empirically disentangle the effect of self-selection regarding family/work orientation, we compare individual cumulative childcare leave lengths to workplace averages. Employers can relate mothers’ leave lengths to the workplace average and decide on their career progress as more information on their family/work orientation is revealed.

Our results show that the differences in outcomes between families of different parities are relatively small, while leave lengths related to workplace averages have large effects. However, the choices mothers make regarding parity and leave length are related to many factors, i.e. endogenous. To account for endogeneity, we use an instrument that indicates if the mother lives in a municipality offering a supplement to home care allowance when the child is born. Municipal supplements offer exogenous variation in leave lengths. Mothers living in a municipality offering the supplement when the child is born are inclined to use longer childcare leaves, even longer than their workplace averages. By applying the instrument, we are able to distinguish the outcomes between never-takers, always-takers, and compliers. Never-takers self-select into shorter leaves than their workplace averages, even if offered municipal supplements which is rewarded. Always-takers self-select into longer leaves than their workplace averages even if they are not offered any supplement which is penalised. The earnings trends of mothers complying with varying replacement levels but deviating from workplace averages experience more modest penalties. The difference between these groups is the LATE IV estimate of the extended leave due to the municipal supplement. The extended leave due to higher supplements aggravates the child penalty by around 4 percentage points.

Mothers with shorter(longer) than workplace average childcare leaves experience lower(higher) penalties. These results are in line with results by Johnsen et al. (2023), who demonstrate that longer childcare leaves among fathers are not penalised if they are in par with the leave lengths of co-workers. Our contribution in this paper is to show that self-selection of childcare leave length has a clear impact on earnings post-birth. In addition, we show that family policies can, indeed, affect the child penalty and the gender gap.

The presented results show the importance of child-care leave length, but cannot rule out that long childcare leaves can also lead to human capital depreciation or lack of accumulation. The outcomes of the two groups of mothers staying home with their children shorter, respectively, longer than their workplace averages but distinguished by the instrument differ. These differences in outcomes cannot be explained by human capital depreciation only. Another possible explanation to our results is that child penalties are consistent with discrimination by employers, for recent arguments, see e.g. de Linde Leonard and Stanley (2020), Yu and Hara (2021). However, child penalties do not necessarily imply that employers discriminate against mothers. Longer childcare leaves are more costly to employers (Ginja et al., 2023). Hence, employers may react to the information they receive regarding mothers’ family/work orientation. As a consequence, the careers progress slower among family-oriented mothers and faster among work-oriented mothers.

In a context where childcare is available, but mothers have the possibility to choose their childcare leave lengths, the individual choices contribute to heterogeneous earning outcomes post-birth, especially where the norm is that mothers take care of their young children. Even if employers expect mothers to stay home with their children during maternity and parental leaves, leave lengths deviating from workplace averages have consequences for their earnings trends. The heterogeneous earnings outcomes contribute to heterogeneous child penalties and gender differences. Hence, public support for individual choices of childcare leave lengths, and specifically long childcare leaves, contradicts gender equality in the labour market.

Previous research show that extensions to relatively short parental leaves are beneficial for the mother and child, but extensions to longer leaves than 1 year may have negative consequences (Canaan et al., 2022; Chuard, 2023). If policymakers are concerned about the child penalties and the consecutive gender inequalities, the option to choose long leaves could be limited to shorter periods than 3 years in the Finnish context. Alternatively, the replacement rate for long leaves could be staggered, i.e. higher in the beginning but reduced step-wise, to encourage a faster return to the labour market (see e.g. Österbacka & Räsänen, 2022). Other policy measures to shorten mothers’ long leave lengths can be reached by introducing longer paternity leave quotas. In addition, supply of affordable and high-quality daycare supports parental labour market participation. However, as of date, evidence of the impact on child penalties and gender equality of these policy measures remains inconclusive (see, e.g. Canaan et al., 2022; Kleven et al., 2024).

## Figures and Tables

**Fig. 5:**
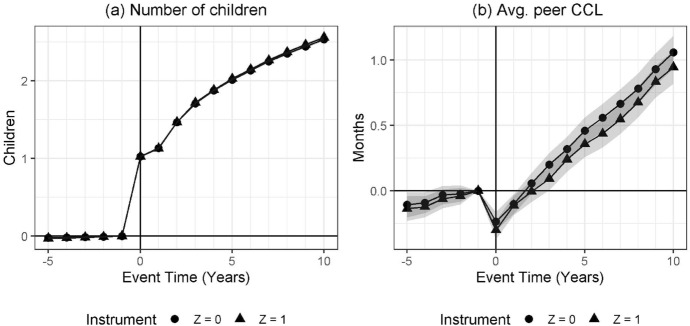
Exclusion restrictions. *Note*: In both panels, the outcomes are estimated separately for a sample where $Z_0 = 0$ and $Z_0 = 1$, respectively. The panels show event time estimates of **a** number of children; **b** change in average childcare leave among peers (where the mother is excluded). In both panels the trend is compared to the average at $t - 1$.

**Fig. 6:**
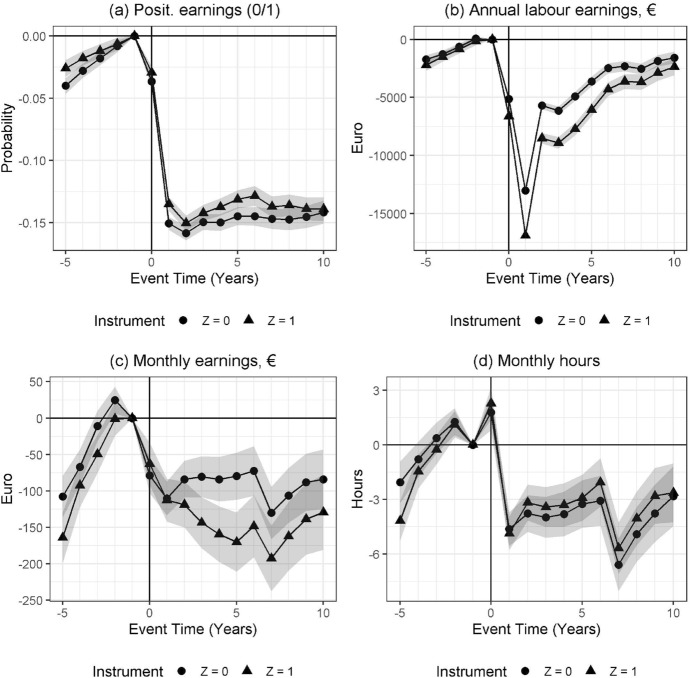
Reduced form results. *Note*: In all panels, the outcomes are estimated separately for a sample where $Z_0 = 0$ and $Z_0 = 1$, respectively. The panels show event time estimates compared to $t - 1$ of **a** probability of positive earnings (all women); **b** annual earnings (all employed); **c** monthly earnings (all employed); and **d** monthly hours (all employed).

**Fig. 7:**
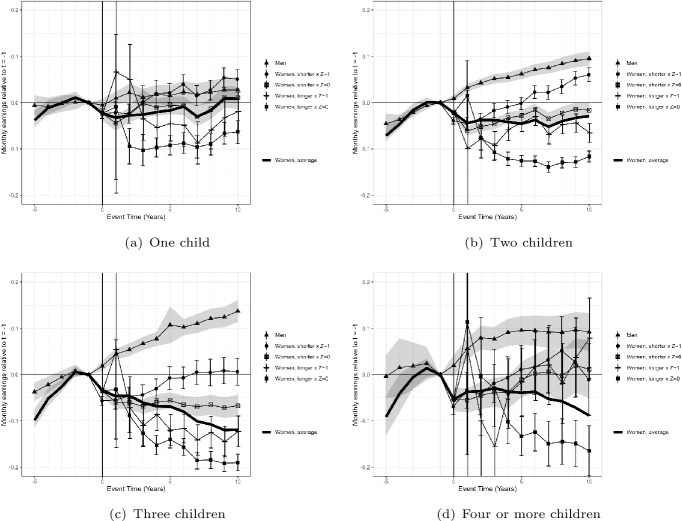
Monthly earnings, ranging between 5 years prior to and 10 years after the first birth, related to monthly earnings pre-birth $(t-1)$ among fathers and mothers, where post-birth events among mothers are interacted with an instrument, $Z_{t=0}$. *Note*: The instrument, $Z_{t=0}$, indicates if the mother’s home municipality offers a home care allowance supplement when the first child is born. The line with triangles represents fathers; the black line represents average mothers; the line with dots represents mothers with equally long or shorter childcare leaves than their workplace averages with municipal supplements (never-takers); the line with crosses represents mothers with equally long or shorter childcare leaves than their workplace averages without municipal supplements (compliers and never-takers); the line with dashes represents mothers with longer childcare leaves than their workplace averages with municipal supplements (compliers and always-takers); the line with squares represents mothers with longer childcare leaves than their workplace averages without municipal supplements (always-takers).

## Data Availability

The data that support the findings of this study are available from Statistics Finland, but restrictions apply to the availability of these data, which were used under licence for the current study, and so are not publicly available.

## Appendix A

See Figs. 5, 6, 7, 8, and 9.

## Footnotes

1 In the sample used, the mean monthly earnings pre-birth among mothers is below 2400 Euros and among fathers around 3000 Euros (presented in Table 1.) For an average family, the monthly day care fee for the first child is below 4% of family gross earnings pre-birth. If the mother receives home care allowance, their gross earnings are reduced by 35–39%. For a single mother with average earnings, the corresponding numbers are 8% and 79–88%, respectively. The earnings-related parental leave is usually based on yearly taxed earnings prior to birth. The replacement rate is 70% up to a ceiling after which it is reduced to 40%, and for even higher earnings to 25%. (These income ceilings change over the years. There are not many mothers surpassing the first ceiling and even fewer the second.)

2 Overall, the SES includes all public sector employees and a majority of private sector employees in employment during the last quarter of the year. Employees from firms with less than five employees, non-organised employees from small firms, company management, firm owners, and their family members are under-represented in the data.

3 If the workplace does not employ women with previous childcare leave histories, then the annual industry average at NACE two-digit level is used. Further, if the two-digit level is not available, we use the one-digit level.

4 Tô (2018) is, to our knowledge, one of the first to consider the impact of leave duration in an event study approach. She splits leave length by quarters related to all mothers in the same birth year and compare their outcomes.

5 Using the instrument at $t=1$ to $t=10$ would generate endogeneity problems since the instrument (*municipal supplement*) causes mothers to increase their childcare leave length, affecting employment (and possibly earnings) directly.

6 These estimates are smaller than those found in Sieppi and Pehkonen (2019), who estimates a long-run child penalty of 25% in Finland, since our sample consists of working parents only who are included in the SES. Fathers out of the labour market dampen their earnings trend, while mothers out of the labour market enlarge their child penalty.

7 Among these two groups, the share of compliers in the sample is uncertain. By estimating the share of compliers in the sample using the method suggested in Angrist and Pischke (2008, pp. 171–172), mothers who switch from shorter than average to longer than average childcare leaves when the treatment status is changed from $Z=0$ to $Z=1$ comprise 5% of the sample. Since a large share of municipalities already offer supplements in 2002, we would expect the share of compliers to be higher than the approximated lower bound of 5% but below 52.4% as shown in Table 3.

8 When the results presented in Fig. 4 are estimated by parity, the results show that the relevance of the instrument for one child and four or more children families is weak, while they are in agreement with the average among two and three children families. These results are not shown here, but available upon request.

9 Sieppi and Pehkonen (2019) estimate that the long-run child penalty for all mothers in Finland is around 25%.
